# Fatigue Life for Different Stress Concentration Factors for Stainless Steel 1.4301

**DOI:** 10.3390/ma12223677

**Published:** 2019-11-08

**Authors:** Przemysław Strzelecki, Adam Mazurkiewicz, Janusz Musiał, Tomasz Tomaszewski, Małgorzata Słomion

**Affiliations:** 1 Mechanical Engineering Department, University of Science and Technology, Kaliskiego 7 Street, 85-789 Bydgoszcz, Poland; adam.mazurkiewicz@utp.edu.pl (A.M.); janusz.musial@utp.edu.pl (J.M.); tomasz.tomaszewski@utp.edu.pl (T.T.); 2 Faculty of Management, University of Science and Technology, Kaliskiego 7 Street, 85-789 Bydgoszcz, Poland; malgorzata.slomion@utp.edu.pl

**Keywords:** fatigue design, S–N curve, high-cycle fatigue, stainless steel

## Abstract

This paper presents the results of the static tensile and fatigue life tests under rotating bending of round 1.4301 (AISI 304) steel samples. The fatigue tests were carried out on smooth and notched samples with three different rounding angles with a shape factor of 1.4, 2 and 2.6. A fatigue life was determined for samples with different shape factors subject to identical loads. The results showed that the scatter of fatigue test results decreases with an increase in shape factor. To evaluate the cracking properties (cracking mode and mechanism), microstructure and fractographic tests of the fractured samples were carried out.

## 1. Introduction

The use of stainless steel in engineering has grown significantly in recent years. In 2018, the use of stainless steel in the industry has grown over 60% compared to 2010 [1], estimated at 50.7 Mt [2]. Due to the common use of this material in engineering, tests to evaluate its structure and properties are crucial. As a result, several articles on its properties have been published [3,4,5,6,7]. Since the material is often used in mechanical engineering, evaluation of its basic mechanical properties, including tensile strength and fatigue life, is crucial.

To determine the fatigue life of a material, an S–N curve (stress vs. number of cycles) is often determined for the smooth samples and a stress concentration factor is calculated to correct the results for a specific structural component [8]. S–N curves can be determined by different models, the comparison of which is presented in [9,10,11]. The last square method is the most simple and commonly used method to estimate model parameters and it is presented in standard [12] and [13]. Other methods, such as the maximum likelihood method [14,15,16] and bootstrap method [17], are also used. Recently, a new method to calculate the fatigue strength for different geometries was presented by Ai et al. [9]. Some of the authors also conducted tests with stepwise increasing amplitude [18]. To increase the reliability of the estimated fatigue life, a reliability factor is also determined depending on the required probability of failure [8,12,19]. The procedure assumes a constant scatter of results for each geometry of the tested component; however, no such relation was shown by the researchers.

A study [20] (pp. 377–380) by Schijve presents the tests carried out on two structural components in which the fatigue life at 50% failure probability was 8.5 times higher for two different components. For the same components, at the failure probability of 1%, the difference in fatigue life was 2.3 times.

The study aimed to verify whether the scatter of fatigue test results varies depending on the stress concentration factor. The 1.4301 stainless steel samples, a material commonly used in mechanical engineering, were used as a test material. The test results showed that the scatter of fatigue life depends on the geometry of the components.

## 2. Materials and Methods

**Figure 1:**
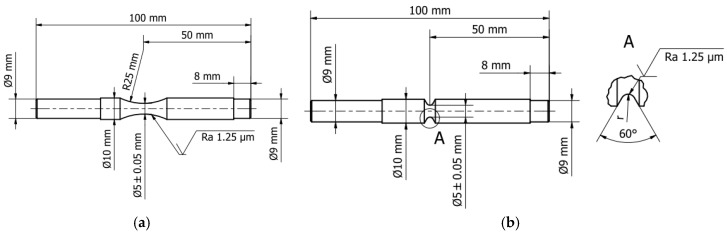
Geometry of the specimen for fatigue testing (**a**) Smooth; (**b**) Notched.

Austenitic 1.4301 (AISI 304) stainless steel was used in the tests [2]. The chemical composition of steel was measured by using a spectrometer, the Oxford Instrument Foundry-Master UV (Oxford Instrument, Abingdon, UK), which fulfils the standard requirements [21] and is shown in Table 1. The samples were made from a 10 mm drawn bar in a raw state, with the dimensions consistent with the standard requirements [22]. The drawing was made at room temperature. The smooth samples for fatigue tests were prepared in accordance with Figure 1a, and the notched samples were prepared in accordance with Figure 1b with the dimensions specified in Table 2. 

**Figure 2:**
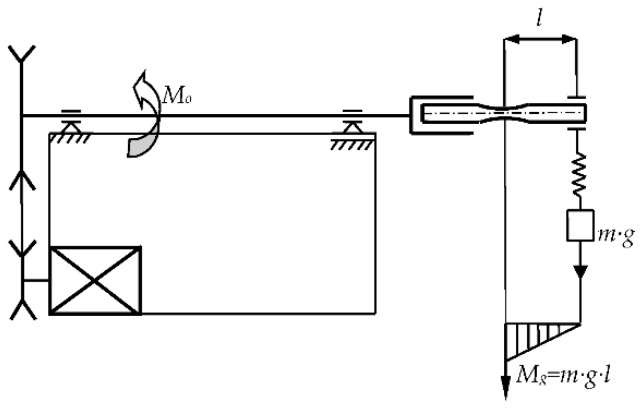
Scheme of test equipment for rotating bending.

A static tension test was carried out on an Instron 8874 testing machine in accordance with the standard requirements [23]. The strain rate was 0.0198 mm/s, which is similar to that presented in [24]. A rotating bending machine described in [25] was used in the fatigue tests. Figure 2 shows the test stand diagram. On one side, the sample was attached to the test stand shaft via a clamp and on the other side a bearing with a load *m* was mounted. The shaft speed *Mo* was 3000 min^−1^ at a loading frequency of 50 Hz. The bending moment *Mg* was equal to the load *m* suspended on the bearing multiplied by the distance between the bearing center and the point of minimum cross-section *l*, which was 46 mm. A load weighing between 5 kg and 13.8 kg was applied. A rotating bending test was carried out in accordance with the standard procedure [26]. The measuring accuracy was 1.15% with a permissible accuracy of 1.3% [26].

## 3. Results

**Figure 3:**
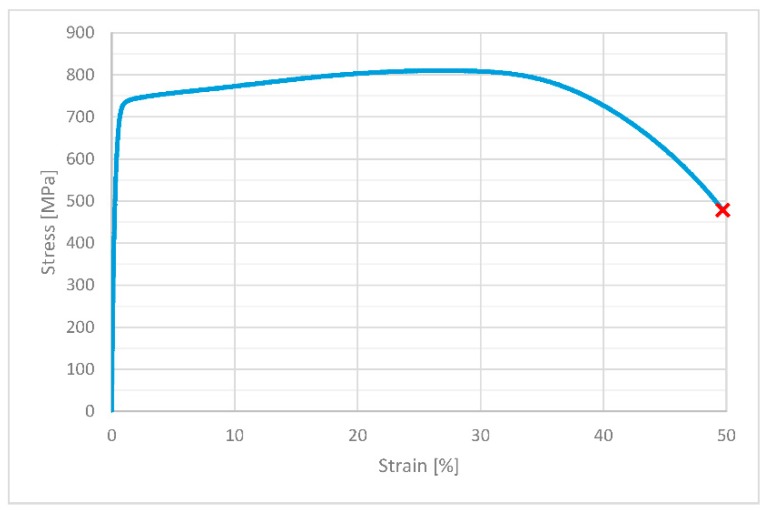
Stress–strain curve for static load for exemplary specimen.

Table 3 shows the mechanical properties calculated based on an axial tensile test at a constant loading rate. The table shows the average values calculated for 20 tests. Figure 3 shows an example stress–strain diagram for the test. The values for yield strength and ultimate strength are higher than the minimum requirements in [27] which should be least 350 MPa and 700 MPa for *S_y_* and *S_u_*, respectively. It should be mentioned that the requirements for plate steel according to [28] are of a lower value, with a minimum of 230 MPa and 540 MPa for yield strength and ultimate strength, respectively. Mechanical properties are different for drawn and plane bars because a bar has high plastic strain after drawing.

**Figure 4:**
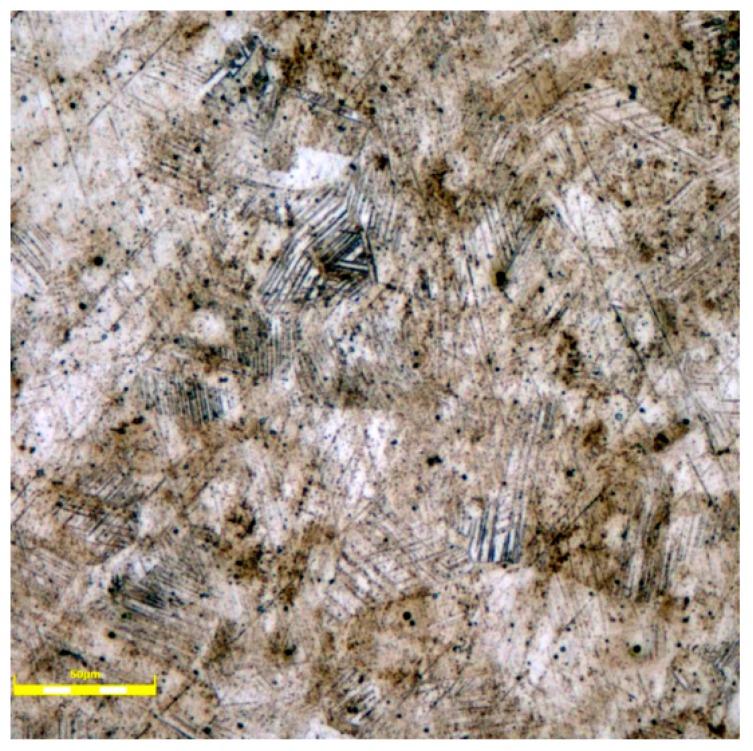
Microstructure of the 1.4301 stainless steel, transverse section in etched.

The microstructure was tested in a cross section perpendicular to the axis of the sample without a load using a scanning digital microscope, the OLYMPUS LEXT OLS4100 Laser Confocal Microscope (Olympus, Tokyo, Japan). Figure 4 shows the example image. Austenite grains are dominant in the visible structure and have sizes between 20 μm and 50 μm. The mean size of the grains was 38.3 μm and the standard deviation was equal to 8.5 μm. The calculation was made by ImageJ software version 1.52a on four images, taken from the scanning digital microscope OLYMPUS LEXT OLS4100, according to standard [29]. A small number of martensite grains (approximate 6%) can also be observed.

**Figure 5:**
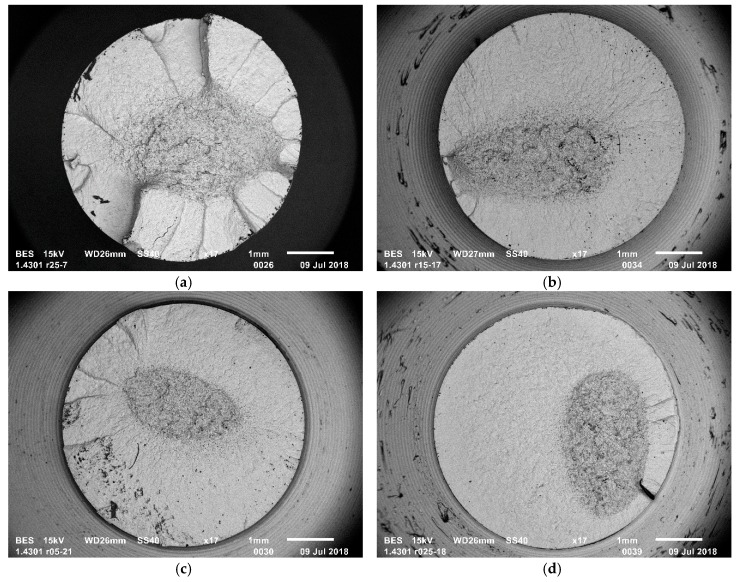
Fatigue fracture of specimen (**a**) smooth; (**b**) notched *r* = 1.5; (**c**) notched *r* = 0.5; (**d**) notched *r* = 0.25.

A fractography of fatigue fractures was carried out for each sample geometry. Figure 5 shows the example fracture images. The tests were carried out on a JEOL JSM-6610 Series Scanning Electron Microscope (JEOL, Tokyo, Japan). The study aimed to compare the propagation of the fatigue cracks for different sample geometries. Figure 5a shows the fracture of a smooth sample. It differs from the fractures in the notched samples, which are often used in fatigue tests. In smooth samples, the crack propagates from several points around the entire sample perimeter, as seen in Figure 5a. In notched samples, the crack is initiated in one or two points, as seen in Figure 5b–d. The results are consistent with the results presented in [30] (p. 39).

**Figure 6:**
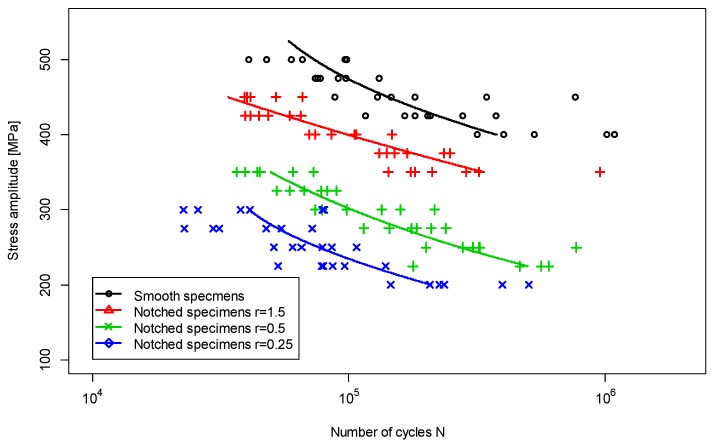
S–N curves for 1.4301 steel.

Figure 6 shows the relationship between fatigue lives and applied nominal stresses. The number of samples and the load range were determined in accordance with [13]. A minimum of 30 tests at five different load levels, i.e., six samples for a single load, were carried out for each sample geometry. The load levels were selected to obtain the fatigue life between 2 × 10^4^ and 10^6^ cycles. 

A 3-parameter Weibull distribution [31] was used to plot the regression line:

(1) $$f\left(N\right)=\frac{\alpha_{v}}{\beta \left(S_{i}\right)}\cdot \left(\frac{N_{i}-\xi \left(S_{i}\right)}{\beta \left(S_{i}\right)}\right)\exp\left[-{\left(\frac{\left(N_{i}\right)-\xi \left(S_{i}\right)}{\beta \left(S_{i}\right)}\right)}^{\alpha_{v}}\right],$$

where:

*α_v_*  the shape parameter,
*β* (*S_i_*) the scale parameter 10*^m^*^⋅^^log(*S*)+*b*^,
*ξ(S_i_)*  the location parameter 10*^n^*^⋅^^log(*S*)+*d*^,
*b*  constant term in an S–N curve equation,
*d*  constant term in an S–N curve equation in the location parameter,
*m*  scale coefficient in an S–N curve equation,
*n*  scale coefficient in an S–N curve equation in the location parameter.

**Figure 7:**
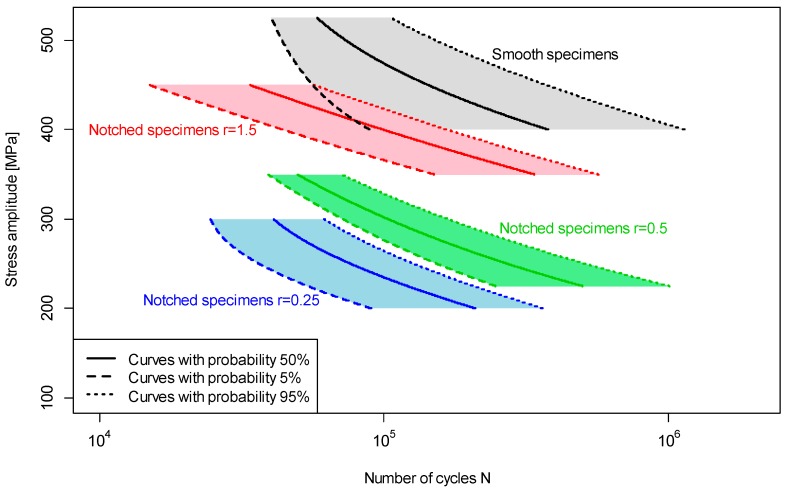
S–N curves for 1.4301 steel with 5% and 95% probability of failure.

Table 4 shows the values of the coefficients estimated using Equation (1). A maximum likelihood method was used to determine the Equation (1) parameters. A method used to determine the S–N curve was discussed in [15,16,32,33,34]. The calculations were carried out using R software version 3.5.1 [35]. Figure 7 shows the S–N curves for 5% and 95% failure probability to highlight the different ranges of scatter of test results for different sample geometries. A solid line shows the S–N curve at 50% failure probability.

To verify the scatter of fatigue life, the coefficient of variation was calculated and expressed following [15]:

(2) $$V=\frac{\sigma_{x}}{\mu_{x}}\cdot 100\%,$$

(3) $$\sigma_{x}=\sqrt{{\beta (S_{i})}^{2}\cdot \left[\Gamma \left(1+\frac{2}{\alpha_{v}}\right)-\Gamma {\left(1+\frac{1}{\alpha_{v}}\right)}^{2}\right]},$$

where:

*V*  the coefficient of variation
Γ  gamma function
*σ_x_*  standard deviation of the 3-parameter Weibull distribution for 105 cycles
*μ_x_*  expected value of the 3-parameter Weibull distribution for 105 cycles.

**Figure 8:**
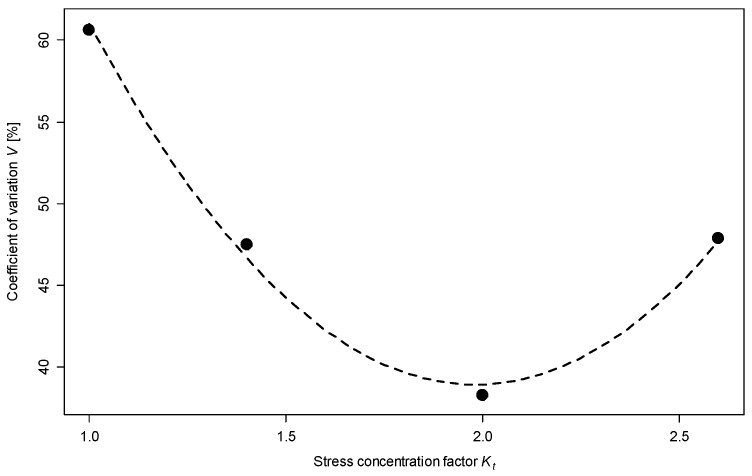
Relationship of variation to stress concentration factor.

Figure 8 shows the calculation results. The graph also includes a regression line plotted using the following equation:

(4) $$V=22.9\;K_{t}^{2}-90.9\;K_{t}+129.$$

## 4. Discussion

The test results show that the scatter of results depends on the sample geometry, as seen in Figure 7 and Figure 8. The test results for 1.4301 stainless steel are similar to the results obtained for AW 6063 T6 aluminium alloy, presented in [36]. The linear regression Equation (4) shows that the lowest scatter of results is observed for the shape factor of 2. A similar value was obtained for AW 6063, as seen in [36]. The notch radius of the specimens was made with an accuracy of 0.01 mm. The precision of the notch radius, with this accuracy of production, is insignificant for smooth specimens and those with a notch radius equal to 1.5 mm and 0.5 mm. However, for the specimen with a radius of 0.25 mm, this accuracy of production influences the stress concentration factor, which can be 2.61 or 2.59 for a radius equal to 0.26 mm or 0.24 mm, respectively. The difference can be ±2 MPa for theoretical stress in the root of the notch for nominal stress equal to 200 MPa.

The test results are crucial in the design of new machine components. The required failure probability of the bogie frame of a railway vehicle must not exceed 5% in accordance with [37]. A lower reliability coefficient can be calculated using Equation [8] (pp. 179–180) and a variance calculated from Equation (4), to reduce the amount of material required.

The scatter of fatigue life is lower for notched specimens because smooth specimens have a larger, highly stressed volume. This phenomenon can be explained by the weakest link theory presented by Weibull [38]. 

The change in fatigue life scatter was also observed when using a salt solution. The test results showing the effects of a corrosive agent on fatigue life and scatter of results for low-carbon 0.26% C steel are presented in [39]. For smooth samples in the air at room temperature, the fatigue life determination error was 4%, whereas, for the samples in 3.5% KCI and 5.5% KCI solution, the error was 1% and 1.5%, respectively. It clearly shows that each factor affecting the fatigue life also affects the scatter of results—a fact verified in the studies, in which the authors attempted to explain the scatter of fatigue life results by the presence of material defects, e.g., [40,41,42,43,44]. The studies correlate the fatigue life scatter with a random size and distribution of material defects.

## 5. Conclusions

The following conclusions can be drawn based on the results:

- notched samples feature lower fatigue life scatter than smooth samples,
- increase in the stress concentration factor decreases the scatter of results,
- an increase in the scatter of fatigue life was observed for samples with *K_t_* = 2.6, explained by the higher sensitivity of notched samples to the accuracy of sample preparation techniques,
- which can be further explained by a different load gradient for a specific sample geometry, which in turn should be verified in further studies.

## Figures and Tables

**Table 1:** Chemical composition of austenitic steel 1.4301 (X5CrNi18-10) according to [21] and measured value.

*%* by Mass							
Item	C	Si	Mn	P	S	Cr	Ni
Standard requirements [21]	≤0.07	≤1.00	≤2.00	≤0.045	≤0.015	17.5–19.5	8.0–10.5
Measured value	0.05	0.75	1.56	0.043	0.010	18.1	9.7

**Table 2:** Dimensions of the notched specimens.

No.	*r* [mm]	*K_t_* [-]
1	1.5	1.4
2	0.5	2
3	0.25	2.6

**Table 3:** Mechanical properties for the 1.4301 steel.

*E* [MPa]	*S_u_* [MPa]	*S_y_* [MPa]	*A* [%]	*Z* [%]
206 553	803	561	78.3	43.8

**Table 4:** Value of linear regression parameters for the 1.4301 steel.

Geometry of Specimen	*r*, [mm]	*α_v_*	*m*	*b*	*n*	*d*
Smooth	25	1.21	−10.2	32.1	−1.08	7.52
Notched	1.5	2.32	−9.2	28.9	−2.72	2.24
	0.5	1.27	−7.2	22.6	−3.54	13.57
	0.25	2.16	−4.9	16.7	2.83	−2.89
